# Evaluation of radiotherapy planning approaches for head and neck patients with tumors close to the skin surface

**DOI:** 10.1002/acm2.14473

**Published:** 2024-07-19

**Authors:** Julie S. Kirk, Carl G. Rowbottom

**Affiliations:** ^1^ Medical Physics Department The Clatterbridge Cancer Centre NHS Foundation Trust Liverpool UK; ^2^ Department of Physics University of Liverpool Liverpool UK

**Keywords:** optimization bolus, radiotherapy planning, virtual bolus

## Abstract

**Purpose:**

In radiotherapy of the head and neck (H&N) it is common for the clinical target volume (CTV) to extend to the patient's skin. Adding a margin for set‐up uncertainty and delivery creates a planning target volume (PTV) that extends beyond the patient surface. This can result in excessive fluence being delivered to the build‐up region and therefore the skin. This study evaluates four different planning methods used when inverse‐planning H&N radiotherapy treatments with CTV extending to the skin. The aim of the study was to determine which planning method gives superior plan quality.

**Method:**

Ten H&N cancer patients with a CTV contoured to the skin were inverse‐planned using four planning methods. The planning methods compared were: cropping the optimization PTV back from the skin surface by 5.0, 3.0, and 0.0 mm and a virtual bolus method. For each planning method, the increased fluence at the skin surface was analyzed. The CTV coverage and skin doses were compared. Plan robustness was evaluated by applying an isocenter shift of ±3.0 mm in the major axes.

**Results:**

The planning method cropping the PTV 0.0 mm from the skin surface results in an increased fluence in the build‐up region. The average volume of CTV receiving 98% of the prescription dose was 89.6% ± 3.4%, 91.6% ± 2.4%, and 93.5% ± 1.7% when cropped 5.0, 3.0, and 0.0 mm, respectively, and 93.4% ± 2.1% for the virtual bolus method. Introducing plan uncertainty affects CTV coverage the most when cropping 5.0 mm. When plan uncertainties are considered the methods of cropping 5.0, 3.0 mm, and the virtual bolus method have the same average skin dose within ±0.3%.

**Conclusion:**

This study shows that a virtual bolus planning method results in no increased fluence at the patient's surface, improves CTV coverage, and is the most robust to changes in setup and patient anatomy.

## 1 INTRODUCTION

In radiotherapy of the head and neck (H&N) region tumor control is achieved by irradiating clinical target volumes (CTVs) to a prescribed dose. CTVs for H&N cancers are complex and individual to each patient. In many H&N patients the CTV can extend to the patient's skin. When a margin is added for uncertainties in set‐up and delivery a planning target volume (PTV) that extends beyond the patient surface is created. It is valid to have a PTV that extends beyond the surface of the patient as it is likely the CTV will fall into this volume throughout the treatment course. When creating a non‐inverse plan the PTV coverage outside of the patient is achieved by simply extending the treatment fields to cover the PTV with a flash region. This ensures CTV coverage for all uncertainties. PTV based inverse‐optimization removes low doses within the PTV, even when caused by the build‐up region. This results in excessive fluence being delivered to the build‐up region and therefore the skin. This can result in acute skin toxicity.^1^ Since the introduction of inverse planning a number of solutions have been considered to compensate for this issue.

One solution is to consider the skin as a sensitive structure and remove the structure from the PTV when optimizing IMRT plans.^1^, ^2^ Thomas and Hoole^2^ found that geometric errors lead to inadequate CTV coverage when using this method. This can be considered to be equivalent to cropping the PTV back from the skin surface; a solution that is described in a number of UK and international H&N radiotherapy clinical trials; such as NIMRAD,^3^ PATHOS,^4^ and JAVALIN.^5^

A virtual bolus method was first investigated by Thilmann et al.^6^ for breast treatments using tangential fields. In this solution water equivalent bolus is virtually added to the patient's surface for optimization only. The virtual bolus is removed for the final dose calculation and not used for treatment. Thomas and Hoole^2^ found that this method gave the most superior results when considering CTV coverage for H&N plans. The use of virtual bolus in the planning of breast cancer treated with arc therapy was studied by Tyran et al.^7^ In this study, plans (RayStation v5.0) with and without virtual bolus were compared by evaluating CTV coverage on a CT performed during treatment and as a consequence of modification in the patient's anatomy. The study showed the virtual bolus plan gave an increased CTV coverage compared to the non‐virtual bolus plan. This demonstrates the benefit of using the virtual bolus during inverse planning to compensate for potential changes in breast shape. This solution is also described in UK and European H&N radiotherapy clinical trials; such as NIMRAD,^3^ PATHOS,^4^ and “Best of” trial.^8^

Due to the variation in guidance given in H&N radiotherapy clinical trials a survey of UK radiotherapy centers was carried out as part of this study. This showed that 18 of the 22 centers that responded did not use the virtual bolus planning method. The survey also showed 21 of the 22 centers used the technique of cropping the PTV in from the patient surface. The amount the PTV was cropped from the surface varied between 3.0 and 6.0 mm.

The results from this survey and the guidance for H&N radiotherapy clinical trials demonstrates there is little consensus in what planning method gives the best solution for optimizing in the build‐up region for H&N plans.

In this paper, different planning methods used to compensate for the excessive fluence in the build‐up region while achieving an acceptable plan for all set‐up uncertainties are compared. The method that gives superior plan quality when considering CTV coverage and skin dose is determined.

## 2 METHODS AND MATERIALS

Computer tomography (CT) images of 10 H&N patients were acquired in head to gantry, supine position, with a slice width of 3 mm and field view of 65 cm. At the time of acquiring the CT images all patients had been immobilized in a five fixation point thermoplastic mask, supported by a headrest and vac bag. The gross tumor volume (GTV) and CTVs were defined and contoured by a radiation oncologist. Each patient had two CTVs; a high dose CTV (CTV_high_) which was irradiated to the highest prescribed dose and a low dose or prophylactic CTV (CTV_low_). All selected patients had the CTV_high_ contoured to the skin surface. The dosimetric parameters to CTV_high_ will be reported in this study. Organs at risk (OARs) were automatically contoured by Mirada^1^ DLC Expert, followed by modification, if required and approval by a radiation oncologist. Treatment plans were created using Eclipse 15.6^2^ for a Varian Truebeam linac. In brief, two complementary 6MV coplanar volumetric modulated arc therapy (VMAT) arcs (one counter clockwise, one clockwise) were used. Each arc had a collimator rotation of 30° and 330°, respectively.

Optimization was performed with the Photon Optimizer Algorithm (PRO) v15.6. The PRO algorithm allows the user to set optimization parameters, these are summarized in Table 1.

**TABLE 1:** Summary of parameters set in Photon Optimizer Algorithm (PRO) v15.6.

PRO v15.6 parameter	Function	Setting
Monitor unit (MU) objective	Limits the total number of MUs in a plan and can increase the size of multi‐leave collimator (MLC) apertures. This is considered important in IMRT plans as small MLC apertures may be associated with dosimetric errors between the calculated and delivered dose^9^	250 times the daily prescribed dose (Gy)
Normal tissue objective (NTO)	Defines how the dose falls off outside the PTVs by limiting the dose level and preventing hotspots in healthy tissue	Standard NTO parameters remained the same for each plan. Distance from target border 0.3 cm, start dose 100%, end dose 60%, fall‐off 0.15.
Automatic intermediate dose calculation	Compensates for differences in dose calculated throughout the optimization using the multi‐resolution dose calculation (MRDC) algorithm and the final dose calculation algorithm	On for all plans. For all calculation throughout the optimization a grid resolution of 2.5 mm was used

The final dose calculation was performed with AcurosXB 15.6, with a dose grid size of 1.0 mm. Dose grid size can have a significant impact on the surface dose calculation. It is expected that a 1.0 mm dose grid size results in calculations that closely agree with measurement and gives high accuracy in surface dose.^10^, ^11^

For each patient four plans were created. These plans included cropping the PTVs back from the skin surface by varying amounts and using virtual bolus in the optimization. The creation of these plans is described below and summarized in Table 2.

**TABLE 2:** Summary of four planning methods used in this study.

Plan name	Optimization PTV	Plan normalization
Crop_5.0 mm	PTV_Crop5.0 mm – PTV cropped 5.0 mm from the skin surface	Median dose to PTV_Crop5.0 mm
Crop_3.0 mm	PTV_Crop3.0 mm – PTV cropped 3.0 mm from the skin surface	Median dose to PTV_Crop3.0 mm
Crop_0.0 mm	PTV_Crop0.0 mm – PTV cropped 0.0 mm from the skin surface	Median dose to PTV_Crop0.0 mm
Virtual bolus	PTV_Crop0.0 mm – PTV cropped 0.0 mm from the skin surface	Median dose to PTV_Crop0.0 mm

### 2.1 Initial plan creation

PTVs were created from CTV_high_ and CTV_low_ with a margin of 5.0 mm in all directions. This margin is used by the local institution and reflects the geometric accuracy of the immobilization system and other uncertainties such as delineation. Additional PTVs were created for optimization by cropping the PTVs 5.0 mm internal from the skin surface. This has the effect of creating a negative CTV to PTV margin when the CTV is contoured to the skin surface. Planning organs at risk volumes (PRVs) were created for all serial structures. A margin of 3.0 mm was applied to the spinal cord, brainstem, and mandible.

An additional structure was created to evaluate doses to the skin, this structure has not been used in any optimization. The skin structure is a 2.0 mm thick shell around the patient with the outer surface consistent with the surface of the patient. The dermis layer of the skin is between 0.05 and 1.5 mm, depending on the anatomic location.^12^ A skin thickness of 2.0 mm has been previously used in similar practical work.^13^, ^14^ Clinical trial protocols such as Keynote‐867^15^ define the skin as a 5.0 mm ring contour. In this study, the skin structure is overlapping the CTV. When reporting maximum doses, the thicker the ring the less likely this structure will represent the actual dose to the skin, therefore a 2.0 mm ring was considered more appropriate.

An external body contour used by Eclipse to calculate the dose distribution was extended to include the patient's thermoplastic mask and an additional 5.0 mm in the region of the PTVs.^16^

Clinically acceptable plans were produced for each patient. Each of these plans were individually optimized, planning aims were prioritized in the following order:

- Meet all mandatory PRV constraints (spinal cord, brainstem, mandible)
- High dose PTV coverage
- Low dose PTV coverage
- Non‐critical OAR constraints (e.g., parotids, larynx, oral cavity)
- Other non‐specified normal tissue

All plans were deemed optimal by two experienced planners. Plans were normalized to the median dose of the high dose PTV that had been cropped back 5.0 mm.^17^

For each patient the same optimization objectives were used for all the planning methods.

### 2.2 Creation of plans with PTV cropped back from skin surface

Additional optimization PTVs were created for each patient by cropping the PTV 3.0 and 0.0 mm from the skin surface. Plans were created for each of these optimization PTVs using identical field arrangements and optimization objectives as the initial plan. All plans were normalized to the median dose of the high dose PTV that had been used in optimization, for example, PTV cropped 3.0 or 0.0 mm from the skin surface.

### 2.3 Creation of virtual bolus plan

**FIGURE 1:**
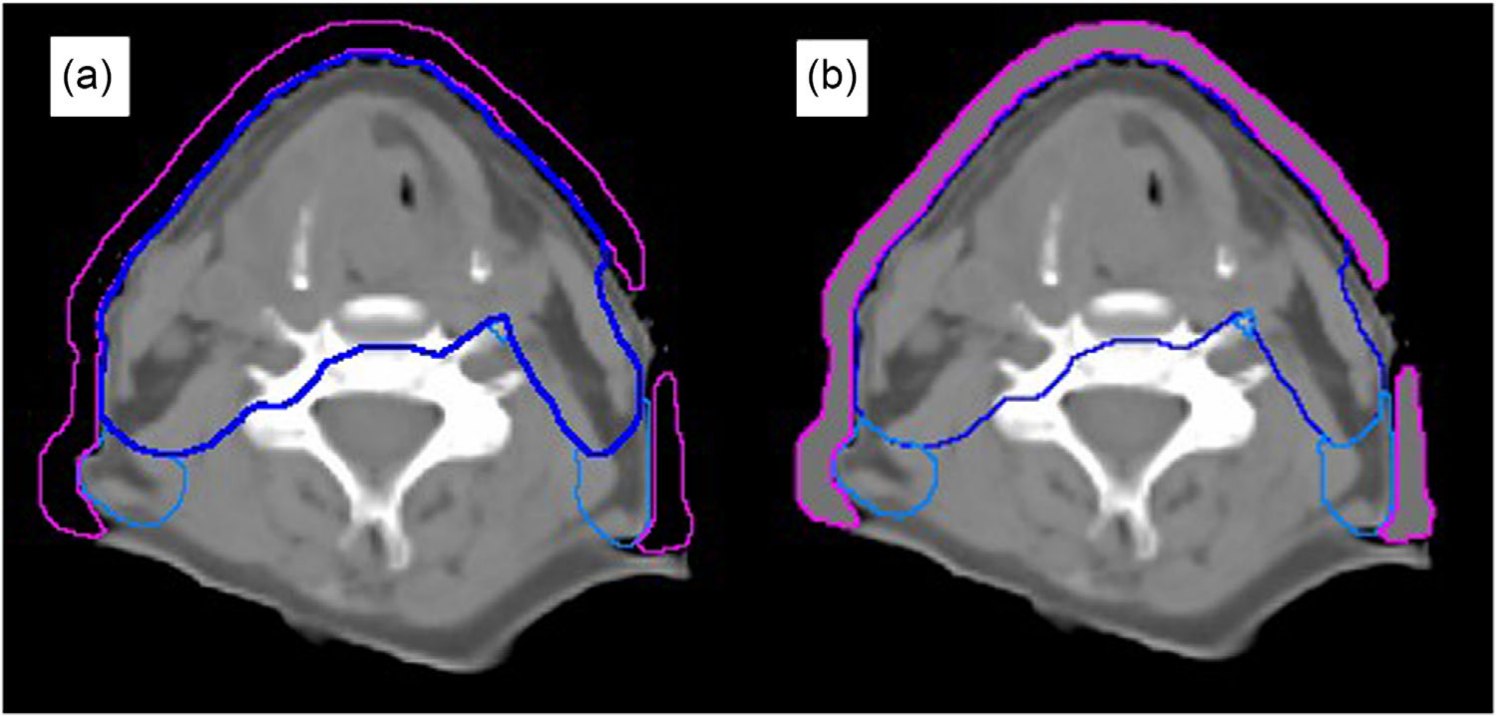
Showing structures required for virtual bolus plan and to check for excessive fluence at the skin surface. Blue structures are the PTVs modified for optimization of the virtual bolus plan. Pink structure is the virtual bolus with a density over‐ride to water for optimization (b) and the over‐ride removed for final dose calculation (a). The over‐ride to water of the virtual bolus (b) is applied to check for excessive fluence at the skin surface.

Plans were created for each patient using the PTV cropped 0.0 mm from the skin surface. These plans were optimized with a virtual bolus structure in place. The virtual bolus structure is a structure that has been created by margining 5.0 mm from the optimization PTV and avoiding the inside of the patient external contour. The PTV and virtual bolus structures are shown in Figure 1. The virtual bolus structure was assigned a material of water for optimization, as shown in Figure 1(b). An identical field arrangement and optimization objectives to the initial plan were used. The virtual bolus structure was removed for the final dose calculation. Plans were normalized to the median dose of the high dose PTV that had been cropped back to the skin surface.

### 2.4 Plan uncertainty

For each plan in each patient the plan uncertainty doses were calculated using the Eclipse plan uncertainty tool. This tool has been used to evaluate plan robustness to shifts in treatment isocenter position. This estimates how the differences in planned patient setup and treated patient setup affect the dose distribution and therefore dose to targets and OARs. The plan uncertainty tool recalculates the original plan with the isocenter shifted by a defined amount in the X, Y, or Z directions. In this study, a shift of 3.0 mm has been applied left, right, anterior, posterior, superior, and inferior to produce six plan uncertainty distributions. A shift of 3.0 mm has been used as this represents the local cone beam CT (CBCT) imaging tolerance across the length of the treatment volume. Although this method of calculating the plan uncertainty shifts the isocenter by 3.0 mm; at the surface it also represents a change in the patient's external contour in a given direction. For example, if the CTV is to the patient's skin surface on the right of a patient; an isocenter shift to the patients left is equivalent to an increase in the external contour on the right. Provided the CTV is still to the patient's skin surface the plan uncertainty distribution will show the CTV coverage change with either a setup uncertainty or change in the external contour at the surface.

### 2.5 Excessive fluence check

Optimizing with a PTV to or beyond the skin surface can result in excessive fluence being delivered to the skin surface.^2^ It is therefore vital to determine if any of the planning methods produces excessive fluence at the skin surface. If excessive fluence is present this will be visible in the dose distribution when the plan is recalculated with a virtual bolus structure added over the PTV. All plans for each patient were recalculated (maintaining planned monitor units) with a water density bolus structure that is margined 5.0 mm from the PTV cropped 0.0 mm from the patients surface and avoiding the inside of the external patient contour, as shown in Figure 1(b). An excessive fluence at the surface will give hotspots within the patient. The dose to 1cc of the entire body (D_1cc_) has been compared between planning methods.

### 2.6 Plan evaluation

For each planning method the dose to 98% of CTV_high_ as a percentage of the prescription dose (D_98%_) has been calculated and compared. Plans have been evaluated by calculating the CTV D_98%_ for the range of plan uncertainties. The purpose of the CTV to PTV margin is to ensure the prescribed dose is delivered to the CTV.^18^, ^19^ A good plan is defined as giving a clinically acceptable coverage of the CTV when shifted by a distance equal to the CTV to PTV margin in any direction.^18^, ^19^ In this study, the isocenter has been shifted by a distance less than the CTV to PTV margin. This shift should therefore give good CTV coverage as the extremes have not been reached. The standard deviation for CTV D_98%_ has been calculated for each planning method across the six plan uncertainties. The standard deviation gives an indication of how robust the plan is to changes in setup and to changes in patient anatomy.

To evaluate the maximum dose to the skin, the dose to 1.0cc and 0.1cc of the skin contour as a percentage of the prescription dose has been calculated for each planning method.

The Wilcoxon signed‐rank test was used to calculate the significance of the dosimetric parameters among the different planning methods.

## 3 RESULTS

### 3.1 Excessive fluence check

**FIGURE 2:**
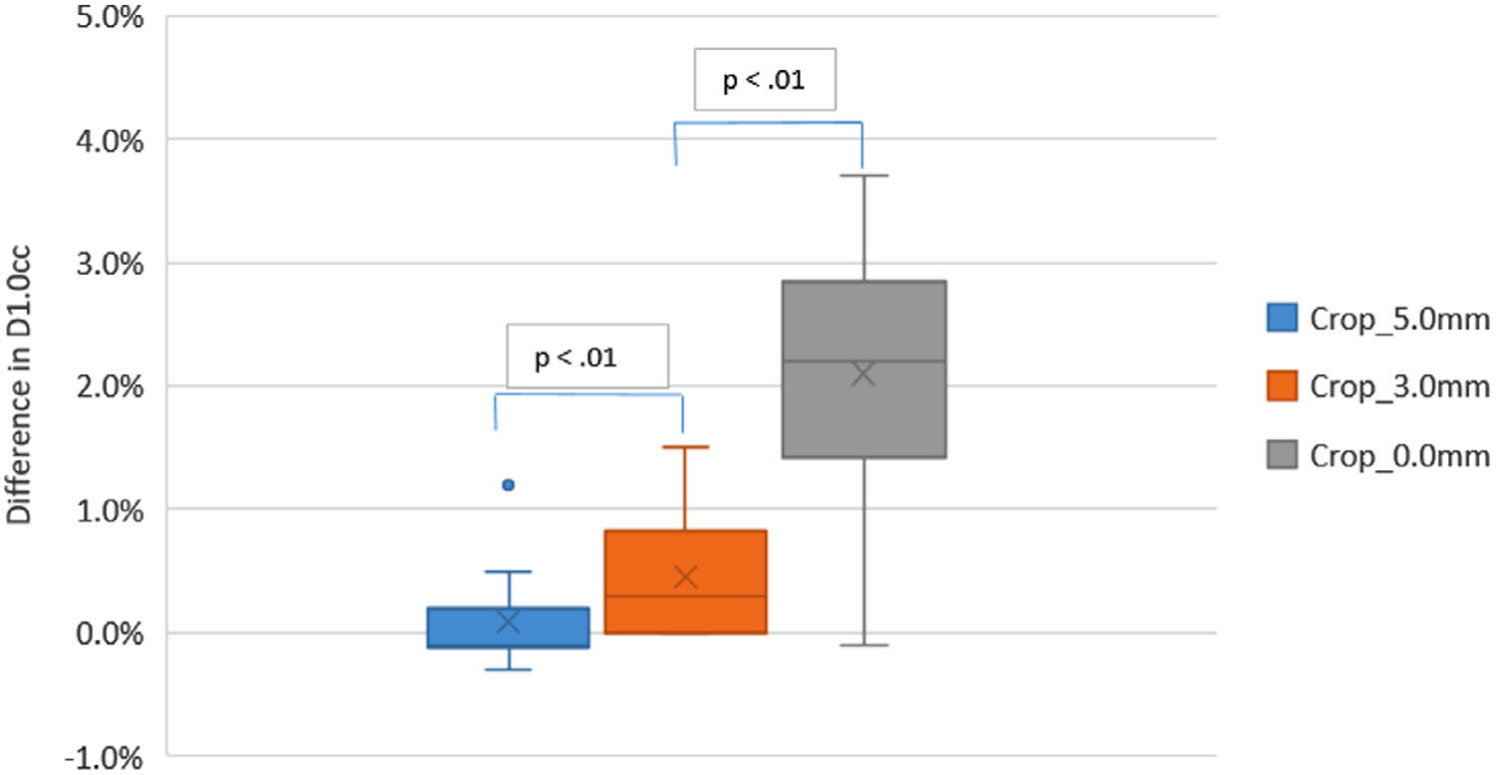
Box plot showing the body dose for the planning methods of cropping the PTV from the skin surface when plans have been recalculated with virtual bolus in situ. The percentage difference from the virtual bolus planning method is shown.

Plans that have been optimized with virtual bolus do not have an excessive fluence at the skin surface. When these plans are recalculated with virtual bolus in‐situ the dose to 1.0cc of the body decreases by an average of 0.4%. Visual inspection of plans from all the datasets also shows no increase in dose at the patient surface. For each planning method the percentage of the prescription dose to 1cc of the body have been compared to the virtual bolus plan. Figure 2 shows the change in body doses for each planning method where the PTVs have been cropped from the skin surface. The average increase in dose compared to the virtual bolus plan being 0.090% ± 0.004%, 0.450% ± 0.005%, and 2.100% ± 0.011% for the PTV cropped 5.0 mm, PTV cropped 3.0 mm, and PTV cropped 0.0 mm plans, respectively.

**FIGURE 3:**
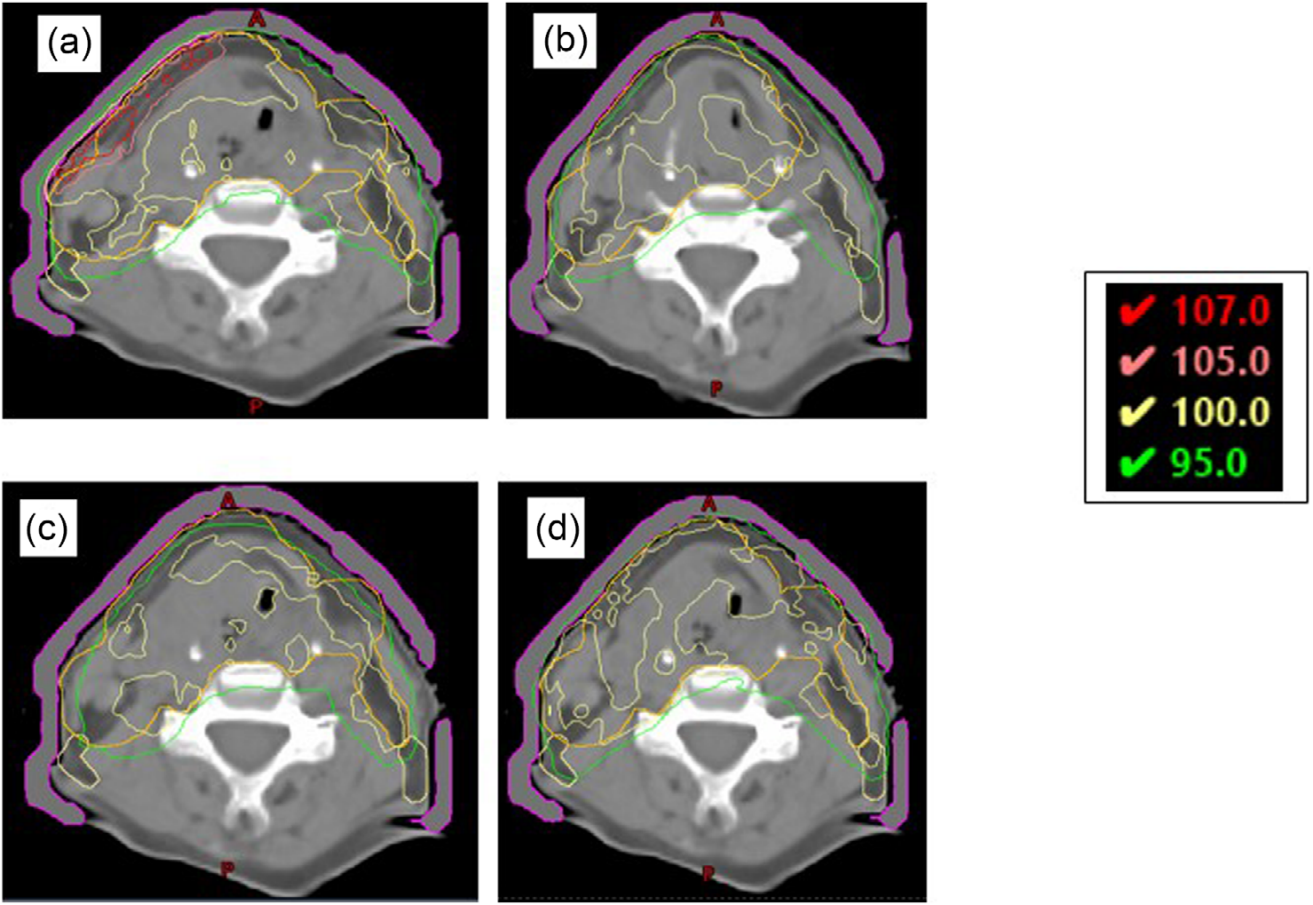
Dose distribution for four different planning methods when recalculated with virtual bolus in situ. The 107%, 105%, 100%, and 95% isodoses are shown for the plans when PTV has been cropped to the skin surface (a), 3.0 mm from the skin surface (b), 5.0 mm from the skin surface (c) and planned with virtual bolus (d). Similar isodoses have been observed on all datasets in this study.

In all cases, the plans with PTV cropped to the skin surface have an increased fluence at the surface. Plans with the PTV cropped back 3.0 mm also give a slight increase in dose within the body contour; however, this is not evident when reviewing the isodoses of the plan. Plans with the PTV cropped back 5.0 mm and the virtual bolus planning method do not show any increase in body dose and therefore it is assumed do not have an increased fluence in the build‐up region. Figure 3 shows the resulting dose distribution of the fluence check for one patient.

When plans are recalculated with virtual bolus the increase in dose is dependent on the amount the PTV is cropped back from the skin surface. As the amount the PTV is cropped back decreases the maximum dose within the body increases.

### 3.2 CTV coverage

**FIGURE 4:**
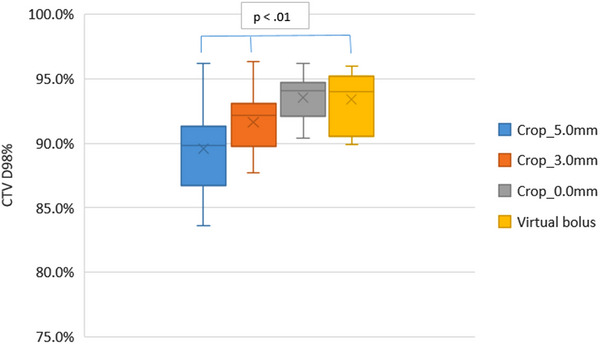
Box plot showing the D_98%_ for the high dose CTV_high_ for each planning method.

For each plan the D_98%_ for each CTV has been calculated. To determine how the D_98%_ is affected by the planning method of cropping the PTV back from the patient's surface or optimizing with virtual bolus, the D_98%_ for each CTV has been compared. The box plot in Figure 4 shows the CTV_high_ D_98%_ for each planning method.

As the amount the PTV is cropped back from the skin surface reduces the D_98%_ increases. For the virtual bolus planning method, the average D_98%_ is the same as cropping 0.0 mm from the patient surface. This is an average increase of 3.8% in the D_98%_ compared to cropping the PTV back 5.0 mm from the patient surface.

The planning method of cropping the PTV 5.0 mm from the skin surface gives on average a CTV_high_ D_98%_ of 89.6% (3.4%). Figure 4 shows for an individual case the D_98%_ reduced to 83.6% when the PTV was cropped 5.0 mm. For this individual case cropping 3.0 and 0.0 mm increased the D_98%_ to 87.2% and 92.0%, respectively. Planning with virtual bolus improved the CTV_high_ coverage from the 5.0 mm cropped plan with a D_98%_ of 89.4%.

### 3.3 Maximum skin dose

The maximum dose to 1.0cc and 0.1cc of the skin has been determined for each plan. This has then been averaged across the 10 patients and is shown in Table 3.

**TABLE 3:** Skin dose for each planning method averaged across the 10 patients.

Planning method	Average dose to 1.0cc skin	Average dose to 0.1cc skin
PTV crop 5.0 mm	93.9% (1.7%) *p* = 0.005	97.3% (1.6%) *p* = 0.007
PTV crop 3.0 mm	95.4% (2.1%) *p* = 0.015	98.4% (1.5%) *p* = 0.051
PTV crop 0.0 mm	98.4% (2.1%) *p* = 0.05	101.4% (1.6%) *p* = 0.017
Virtual bolus	97.3% (2.2%)	99.8% (2.1%)

*Note*: Statistical difference compared to the virtual bolus planning method is shown.

The skin dose increases as the amount the PTV is cropped from the surface reduces; with cropping the PTV to surface giving the highest skin dose.

### 3.4 Plan uncertainty

#### 3.4.1 CTV coverage

The difference in the average D_98%_ for CTV_high_ across the six plan uncertainties compared to the D_98%_ for the non‐shifted plan is shown in Table 4. For all planning methods, the introduction of plan uncertainty on average reduces D_98%_ from the non‐shifted plan. The reduction in the D_98%_ increases as the amount the PTV is cropped back from the skin surface increases. In most cases, the average difference from the non‐shifted plan is less than 1%. This suggests that if all plan uncertainties are random then the delivered CTV D_98%_ will be within 1% of the calculated (non‐shifted) plan for all planning methods.

**TABLE 4:** Difference in the averaged CTV_high_ D_98%_ across the six plan uncertainties compared to the D_98%_ for the non‐shifted plan.

Planning method	1	2	3	4	5	6	7	8	9	10	Av	*p*
**PTV crop 5**.0 mm	−0.9%	−0.5%	−0.5%	−0.7%	−0.3%	−1.2%	−0.9%	−0.3%	−0.6%	−0.1%	−0.6%	0.005
**PTV crop 3**.0 mm	−0.6%	−0.4%	−0.5%	−0.4%	−0.1%	−1.1%	−0.3%	−0.2%	−0.1%	−0.1%	−0.4%	0.021
**PTV crop 0**.0 mm	−0.2%	−0.1%	−0.2%	−0.3%	−0.1%	−0.3%	−0.1%	−0.2%	0.0%	0.0%	−0.2%	0.856
**Virtual bolus**	−0.2%	0.0%	−0.1%	−0.2%	0.0%	−0.4%	0.0%	−0.1%	−0.2%	−0.2%	−0.2%	–

*Note*: Statistical difference compared to the virtual bolus planning method is shown.

The standard deviation for CTV_high_ D_98%_ has been calculated for each planning method across the six plan uncertainties. Table 5 shows the standard deviation for each planning method for each patient.

**TABLE 5:** The standard deviation for CTV_high_ D_98%_ for each planning method across the six plan uncertainties.

Planning method	1	2	3	4	5	6	7	8	9	10	Av	*p*
**PTV crop 5**.0 mm	2.8%	2.7%	2.3%	2.5%	0.6%	3.6%	3.2%	1.8%	2.5%	0.7%	2.3%	0.005
**PTV crop 3**.0 mm	1.5%	1.4%	1.9%	1.0%	0.3%	2.2%	1.2%	1.1%	1.1%	0.3%	1.2%	0.005
**PTV crop 0**.0 mm	0.1%	0.6%	1.1%	0.6%	0.2%	0.5%	0.5%	1.3%	0.7%	0.0%	0.6%	0.161
**Virtual bolus**	0.3%	0.2%	0.4%	0.4%	0.2%	0.5%	0.6%	0.4%	0.3%	0.2%	0.3%	–

*Note*: Statistical difference compared to the virtual bolus planning method is shown.

**FIGURE 5:**
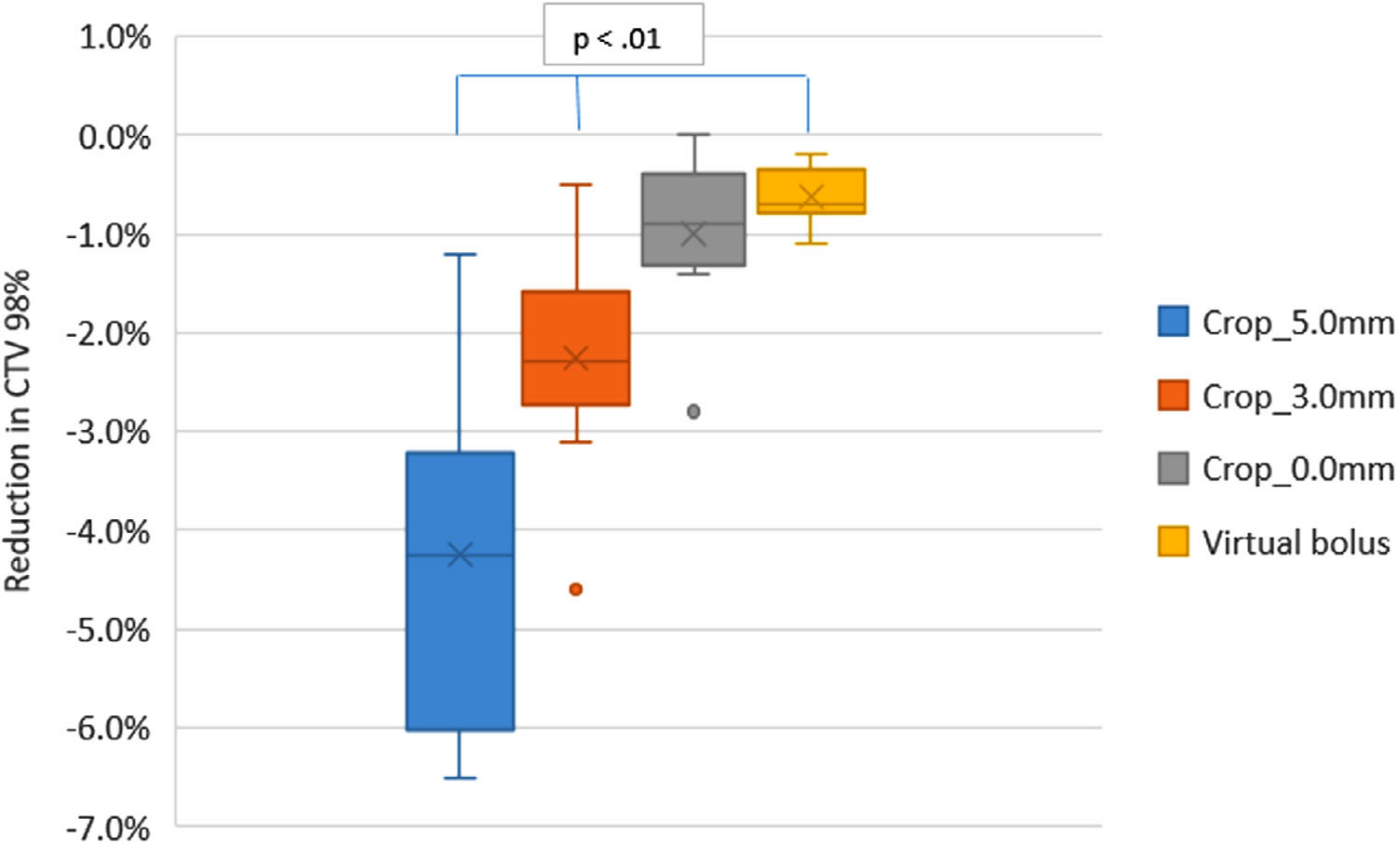
Box plot showing the greatest reduction in high dose CTV_high_ D_98%_ resulting from an isocenter shift of 3.0 mm.

This data shows that the virtual bolus planning method gives the least variation in CTV D_98%_ when a plan uncertainty of 3.0 mm is applied. The variation in CTV D_98%_ increases as the amount the PTV is cropped back from the surface increases. The standard deviation does not give an indication of the direction of change. An increase in the D_98%_ would benefit the distribution, however, a reduction in D_98%_ would indicate further compromise to the CTV and therefore likely reduce the tumor control probability. The greatest reduction in the D_98%_ from the non‐shifted plan for each planning method is shown in Figure 5.

Figure 5 shows that the virtual bolus plans have an average reduction in D_98%_ of 0.6% (0.00%) The reduction in D_98%_ increases as the amount the PTV is cropped back from the surface increases. The planning method of cropping the PTV back from the skin surface by 5.0 mm gives the greatest reduction in CTV_high_ D_98%_ by an average of 4.2% (0.02%)

#### 3.4.2 Skin dose

The plan uncertainty calculations show changes in skin dose when 3.0 mm shifts are applied. Table 6 shows that the greatest increase in skin dose is for plans that have the PTV cropped back 5.0 mm from the skin surface.

**TABLE 6:** Maximum increase in skin D_0.1cc_ for each plan due to 3.0 mm shift in isocenter position.

Planning method	1	2	3	4	5	6	7	8	9	10	Av	*p*
**PTV crop 5**.0 mm	**1.5%**	**1.8%**	**1.4%**	**1.5%**	0.6%	**3.3%**	**1.3%**	**1.4%**	**1.8%**	**1.4%**	**1.6%**	0.007
**PTV crop 3**.0 mm	0.5%	0.5%	**1.6%**	0.9%	0.6%	**3.2%**	0.5%	**1.1%**	0.8%	0.8%	**1.1%**	0.017
**PTV crop 0**.0 mm	0.9%	0.2%	0.9%	0.0%	0.5%	0.5%	0.2%	**1.2%**	0.3%	0.8%	0.6%	0.093
**Virtual bolus**	0.3%	0.2%	0.0%	0.0%	0.8%	0.0%	0.6%	0.6%	0.4%	0.4%	0.3%	

*Note*: Statistical difference compared to the virtual bolus planning method is shown.

The maximum skin dose for each planning method can be determined by adding the maximum increase in dose (Table 6) to the skin dose calculated from the non‐shifted plans (Table 3). The planning methods of using virtual bolus and cropping the PTV 3.0 and 5.0 mm have an average maximum skin dose of 99.8% ± 0.3%. The planning method of cropping PTV 0.0 mm has an average maximum skin dose of 102.0%

## 4 DISCUSSION

### 4.1 Excessive fluence

Inverse planning can result in solutions that give higher fluence to tangential beam segments near the skin surface, in an attempt to counter the low dose in the build‐up region.^2^ Thomas and Hoole^2^ demonstrated that hot‐spots of 126% can be delivered to the skin by plans where PTV is to the skin surface. The increased fluence in the build‐up region has not previously been validated using modern optimization algorithms, such as the PRO algorithm used in this study.

The increase in skin dose observed when bolus added is significantly lower than the 126% reported by Thomas and Hoole.^2^ Thomas and Hoole^2^ used Xio v4.02 (Computerized Medical Systems, St Louis) to produce IMRT plans with five segmented step‐and‐shoot fields. There have been many changes to inverse planning and IMRT delivery since 2004. These changes are likely to have smoothed the fluence of IMRT and VMAT fields and therefore reduce spikes in fluence at the patient's surface.

Results from the fluence check show that plans with PTV cropped to the skin surface have an increased fluence at the surface, and as the amount the PTV is cropped back increases the maximum dose within the body decreases.

### 4.2 High dose CTV coverage

Figure 4 shows that cropping the PTV back from the patient surface for optimization results in a reduced CTV coverage compared to using the virtual bolus method. As the amount the PTV is cropped back from the skin surface decreases, the CTV D_98%_ increases as a result of optimizing to a target that includes more of the CTV.

Plan uncertainty results in Table 5 and Figure 5 demonstrate how a negative CTV to PTV margin results in a plan that is less robust to systematic changes in setup and anatomy. For H&N patients, changes in the patient's anatomy are likely to be systematic, for example, patient weight loss or gain or tumor growth. Cropping the PTV 5.0 mm back from the patient surface could result in a 4.2% reduction in the CTV D_98%_ with just a 3.0 mm anatomy change or systematic setup shift. This results in a further reduction in the CTV coverage than reported in the un‐shifted, intended plan. All the planning methods are subject to a change in the CTV D_98%_ when applying an isocenter shift, this is due to the 0.0 mm or negative CTV to PTV margin in the direction of the skin. Some shifts increase the CTV D_98%_ and are likely to improve the tumor control probability. Other shifts reduce the CTV D_98%_, compromising the CTV further. Any changes in setup or anatomy are unknown prior to treatment therefore plans need to be robust to changes or shifts in any direction.

The virtual bolus planning method and cropping the PTV to the patient's skin produces plans with the best CTV coverage and the most robust plans when compared to the planning method of cropping the PTV from the patient surface. The cropping method of planning removes increased fluence at the patient's surface but reduces the CTV coverage and makes the plan less robust to changes in anatomy and setup uncertainties.

### 4.3 Maximum skin dose

When comparing plans that have not had an isocenter shift applied, the maximum skin dose reduces by approximately 1% for every 1.0 mm the PTV is cropped back from the surface. In some cases, for the virtual bolus planning method and the method of cropping the PTV to 0.0 mm, the maximum skin doses were greater than 100%. However, these plans have not been optimized with a skin objective, with only the maximum PTV objective preventing hotspots within this volume.

Thomas and Hoole^2^ results show an increase in maximum skin dose when uncertainty shifts are applied. The maximum dose to the skin increased to 126% from 115% when a 10.0 mm shift was applied to a plan that had been optimized with the PTV to the patient's skin. Changes to inverse planning and IMRT delivery have improved the fluence at the patient's surface. This removes the need to crop the PTV back from the patient's surface as much as 5.0 mm and makes optimized plans more robust to changes in setup and anatomy.

## 5 CONCLUSION

The fluence check results and maximum skin doses with uncertainty shifts applied show that cropping the PTV to the patient surface does give a spike in fluence when optimizing with Eclipse PRO algorithm V15.6. The fluence check results show the need to crop the optimization PTV back from the surface as much as 5.0 mm has reduced with up‐to‐date optimization algorithms and delivery methods.

Reducing the amount the optimization PTV is cropped back or using a virtual bolus planning method ensures the plan is more robust to changes in patient setup or anatomy, as summarized in Table 7. It is therefore recommended that if the CTV is within 3.0 mm of the patient surface a virtual bolus planning method is used. It is also recommended that if the PTV is cropped back from the patient surface, then the PTV should be cropped back by no more than 3.0 mm.

**TABLE 7:** Summary of the four planning methods used when inverse‐planning H&N radiotherapy treatments with CTV extending to the skin.

Planning method	Increase in surface fluence	CTV_High_ D_98%_	Change in CTV_High_ D_98%_ due to plan uncertainties	Skin D_0.1cc_	Increase in skin D_0.1cc_ due to plan uncertainties
PTV crop 5.0 mm	No	89.6%	−0.6%	97.3%	1.6%
PTV crop 3.0 mm	Minimal	91.6%	−0.4%	98.4%	1.1%
PTV crop 0.0 mm	Yes	93.5%	−0.2%	101.4%	0.6%
Virtual bolus	No	93.4%	−0.2%	99.8%	0.3%

## AUTHOR CONTRIBUTIONS

Julie Kirk contributed to study design, data collection, analysis and interpretation, and drafted the manuscript. Carl Rowbottom contributed to study design, study supervision, and critical revision of the manuscript.

## CONFLICT OF INTEREST STATEMENT

The authors declare no conflicts of interest.

## Footnotes

1 Mirada Medical Systems Ltd, Oxford, UK.

2 Varian, Palo Alto, California, USA.
